# How to implant a phrenic nerve stimulator for treatment of central sleep apnea?

**DOI:** 10.1111/jce.13898

**Published:** 2019-03-18

**Authors:** Ralph S. Augostini, Muhammad R. Afzal, Maria Rosa Costanzo, Randy Westlund, Christoph Stellbrink, Klaus Gutleben, Sanjaya Gupta, Moeen Saleem, Timothy W. Smith, Michael Peterson, Michael Drucker, Andrew Merliss, John Hayes, Christen Butter, Matthew Hutchinson, Dariusz Jagielski

**Affiliations:** ^1^ The Ohio State University Wexner Medical Center Columbus Ohio; ^2^ Advocate Heart Institute Chicago Illinois; ^3^ Respicardia Inc Minnetonka Minnesota; ^4^ Klinikum Bielefeld Bielefeld Delaware; ^5^ Ruhruniversität Bochum Bad Oeynhausen Delaware; ^6^ Department of Cardiology, University of Missouri‐Kansas City School of Medicine Saint Luke's Mid‐America Heart Institute Kansas City Missouri; ^7^ Washington University School of Medicine St Louis Missouri; ^8^ United Heart and Vascular Clinic St Paul Minnesota; ^9^ Novant Health Cardiology Winston‐Salem North Carolina; ^10^ Bryan Medical Center Lincoln Nebraska; ^11^ Marshfield Clinic Marshfield Wisconsin; ^12^ Heart Center Brandenburg in Bernau/Berlin & Brandenburg Medical School Bernau Delaware; ^13^ University of Arizona College of Medicine Tucson Arizona; ^14^ 4th Military Hospital Wroclaw Poland

**Keywords:** central sleep apnea, phrenic nerve, phrenic nerve stimulation, transvenous stimulation

## Abstract

**Background:**

Central sleep apnea (CSA) is a breathing disorder caused by the intermittent absence of central respiratory drive. Transvenous phrenic nerve stimulation is a new therapeutic option, recently approved by the FDA , for the treatment of CSA.

**Objective:**

To describe the technique used to implant the transvenous phrenic nerve stimulation system (the remedē System, Respicardia, Inc).

**Methods:**

The remedē System is placed in the pectoral region, typically on the right side. A single stimulation lead is placed in either the left pericardiophrenic vein (PPV) or the right brachiocephalic vein (RBC). A sensing lead is placed into the azygous vein to detect respiration.

**Results:**

In the remedē System Pivotal trial, 147 of 151 (97%) patients were successfully implanted with the system. Sixty‐two percent of stimulation leads were placed in the PPV and 35% in the RBC. Mean procedure time was 2.7 ± 0.8 hours and 94% of patients were free from implant‐related serious adverse events through 6 months.

**Conclusion:**

In patients with CSA, transvenous phrenic nerve stimulation is an effective and safe therapy with an implant procedure similar to that of cardiac implantable electronic devices.

## 1 BACKGROUND

Central sleep apnea (CSA) is a breathing disorder characterized by periods of apnea or absence of airflow caused by absent central respiratory drive because of increased sensitivity to the partial pressure of CO_2_.^1^ CSA commonly presents as Cheyne‐Stokes breathing, characterized by cycles of deep, rapid, crescendo decrescendo breathing (hyperpnea), followed by slower, shallower breathing (hypopnea), or no breathing without respiratory effort from the diaphragm (apnea).^1^ Etiologies of CSA include cardiovascular disease, stroke, chronic use of opioids, renal disease, and idiopathic causes. The prevalence of CSA in atrial fibrillation and heart failure is as high as 30% and 40%, respectively.^2^, ^3^ Moreover, a well‐established relationship exists between Cheyne‐Stokes respirations and increased mortality in patients with heart failure.^4^, ^5^, ^6^ In addition to increased mortality, the recurring hypoxemia and neurohormonal activation associated with CSA lead to systemic and pulmonary hypertension, arrhythmias, and increased cardiovascular burden.^5^, ^6^ To date, treatment options for CSA have been limited, especially in patients with heart failure and reduced left ventricular function where mask‐based therapies may, in fact, lead to an increase in mortality (SERVE‐HF).^7^, ^8^

The remedē System is a novel implantable technology that is FDA‐approved to treat moderate to severe CSA in adult patients.^9^ The remedē System is comprised of an implantable pulse generator (IPG), a stimulation lead for unilateral phrenic nerve stimulation, an optional sensing lead to detect respirations and a programmer. The IPG is a programmable device that provides an electrical stimulus to the left or right phrenic nerve and monitors the patient's respiration using transthoracic impedance. Stimulation of the left or right phrenic nerve causes the diaphragm to contract, thereby developing negative intrathoracic pressure and restoring regular breathing pattern.^10^

Because of the lack of therapeutic options available for patients with CSA and the morbidity associated with the disease, phrenic nerve stimulation is an important treatment, especially for patients with concomitant heart failure. The remedē System Pivotal Trial demonstrated significant improvements in the number of sleep apnea events, sleep architecture, hypoxia, and arousals (all *P* < 0.001). The safety profile demonstrated a 91% freedom from adverse events associated with the implant procedure, the remedē System, or delivered therapy through the 12‐month visit. Further, the study showed significant improvements in patient's quality of life as measured by the Epworth Sleepiness Scale and Patient Global Assessment.^9^ Recently, improvements were seen at 12 months in ejection fraction, Minnesota Living with Heart Failure Questionnaire, and a trend towards increased time to first heart failure hospitalization.^11^ This article describes the implant techniques and experience of this important new therapeutic modality for the treatment of CSA.

## 2 METHODS

### 2.1 Venous access

The implant procedure is performed in the electrophysiology laboratory under conscious sedation as the patient and physician must communicate during stimulation lead placement testing. Patient preparation and antibiotics are similar to cardiac implantable electronic device (CIED) implants.^12^ The remedē System guide catheter (Respiguide, Respicardia, Inc) and stimulation leads are designed primarily for right‐sided access, however, the system can be implanted on the patient's left side. Venous access is obtained by means of the right axillary, cephalic, or subclavian vein. Two long access wires (150 cm) are advanced into the inferior vena cava.

### 2.2 Implantation of the stimulation lead

Transvenous phrenic nerve stimulation uses a stimulation lead implanted adjacent either the left or right phrenic nerve that has multiple electrodes, which can be programmed in unipolar or bipolar configurations. Depending on patient anatomical characteristics the left or right simulation lead (eg, respistim L, LQ, or LQS [left] lead or the respistim R [right] lead, Respicardia, Inc) is implanted in either the left pericardiophrenic vein (PPV) or right brachiocephalic vein (RBC), which run adjacent to the respective nerve. The left side is preferred because of vessel size and relative proximity to the phrenic nerve. Stimulation is effective on either side with 60% and 53% of patients achieving >50% reduction in AHI with left and right stimulation leads, respectively.^9^

Placement of a left stimulation lead was attempted in all 151 patients enrolled in the remedē System Pivotal Trial (150 respistim LQS Model 4065, 1 respistim LQ Model 5065). The LQ and LQS stimulation lead is a quadripolar, transvenous, over‐the‐wire lead with two IS‐1 terminal pins and four ring electrodes; the LQS has a preformed S‐shape bias at the distal end. Classically, the PPV ostium is located below the left internal jugular vein on the inferior aspect of the left brachiocephalic vein (LBC). The procedures and techniques used to deploy the lead into the PPV are described below.

**Figure 1:**
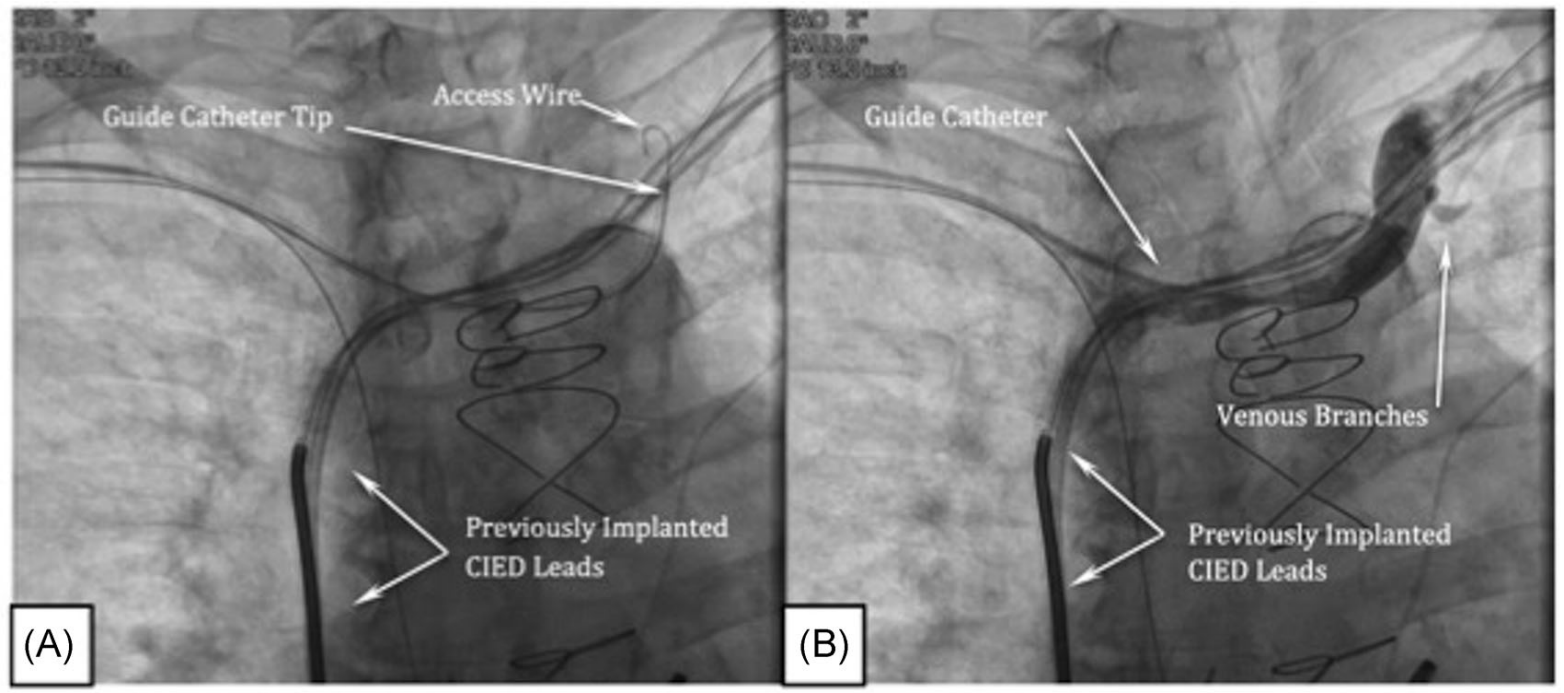
A, Guide catheter at the junction of the left brachiocephalic (LBC) vein and left subclavian veins. B, Nonselective venogram showing branches taking off from the LBC vein

**Figure 2:**
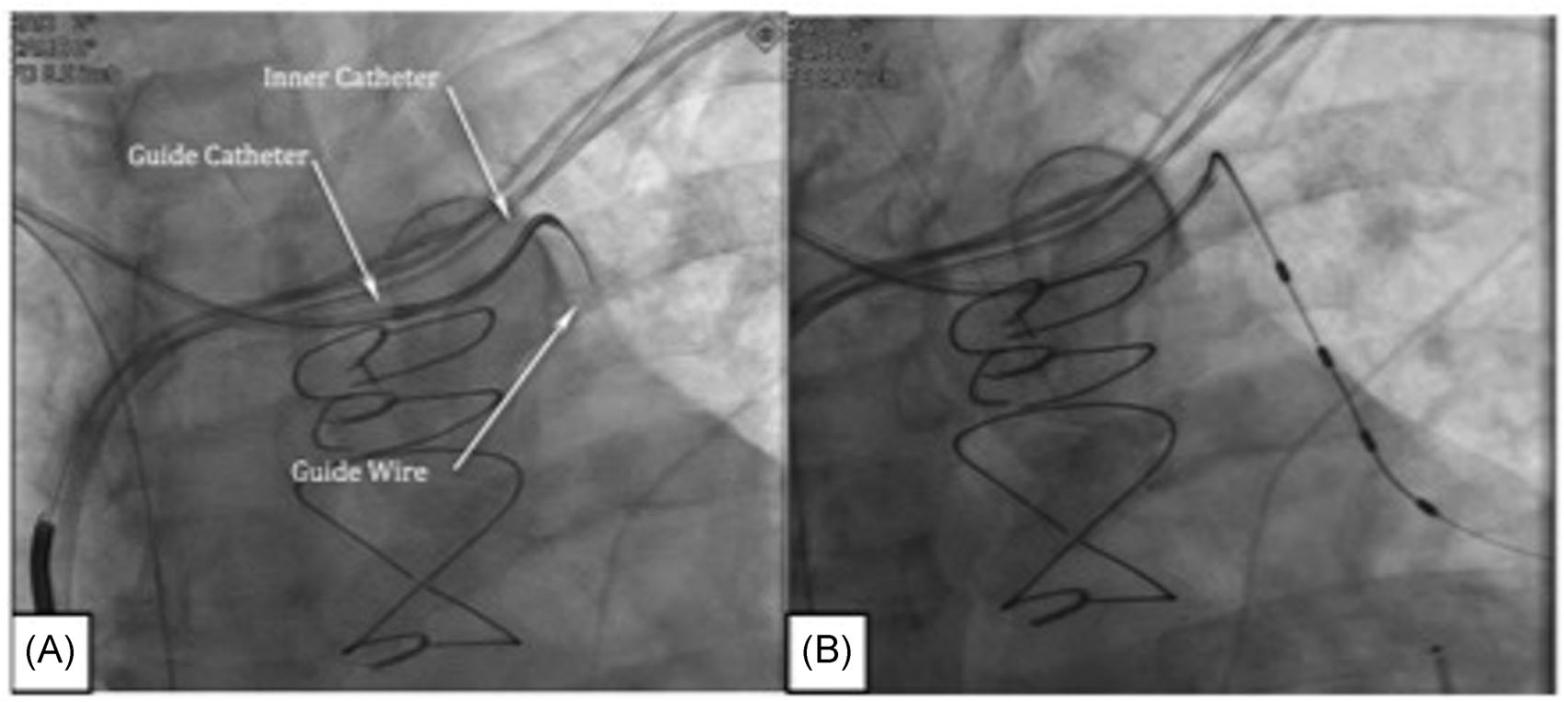
A, Probing with the 0.014” coronary wire and cannulating the pericardiophrenic vein. B, Deploying the left stimulation leads to the level of the left atrium

To visualize the PPV, the 7 French (Fr) guide catheter is delivered over an access wire into the superior vena cava (SVC). Using the guide catheter and the access wire, the guide catheter is advanced into the LBC (Video S1), and the tip is positioned at the junction of the left subclavian and LBC veins (Figure 1A). With the guide catheter and inner catheter oriented towards the floor of the LBC, a nonselective contrast injection is performed to identify venous branches (Figure 1B; Videos S2 & S3). Then, a 5 Fr inner catheter (Merit Medical, Model # 57538CSV‐WOR) is advanced into the guide catheter. The inner catheter telescopes beyond the guide catheter and is oriented towards the LBC floor, the entire assembly is slowly dragged medially while monitoring the catheter tip for deflection into a branch vein. Alternatively, the area below the internal jugular vein is examined by gentle probing with a 0.014” coronary wire (Figure 2A; Video S4) and/or bolus of contrast. Contrast used during this step of the procedure should be undiluted. Visualization of the PPV ostium should be performed cautiously by hand to avoid venous dissection or contrast extravasation, which may render access and visualization of the PPV difficult.

While the PPV ostium is most frequently on the floor of the LBC, some variability exists, and it can be located up to 40 degrees posteriorly or anteriorly. The PPV ranges in size from 3 to 6 Fr.^13^ Using puffs of contrast in the left anterior oblique (LAO) projection, the PPV ostium can be visualized in relation to the internal thoracic and left superior intercostal veins. The PPV runs along the border of the mediastinum and lies within the parietal pericardium until it reaches the cardiac apex. The vein is generally isodiametric along its entire length and is known to have valves 1 to 2 cm inferior to the ostium. The anatomy of the PPV is variable; presentations include a shared common ostium with the internal thoracic vein and a shared common ostium with the left superior intercostal vein.

Once the PPV is visualized and the vessel is accessed using a 0.014” coronary wire, the wire is passed to the level of the cardiac apex. Instructing the patient to make several deep breaths can facilitate advancement of the wire along the length of the PPV (Video S5). Deep breathing expands the thorax and lengthens the PPV, resulting in a momentary reduction of the vessel's tortuosity and increased venous volume that helps to open valves. With the support of the wire placed deep in the PPV and the additional support of the inner catheter, the guide catheter is advanced and stabilized within the PPV ostium. The inner catheter is then removed while maintaining a deep apical position of the coronary wire. The stimulation lead is deployed by back loading the lead onto the coronary wire and navigating the lead through the guide catheter into the PPV to the level of the left atrium (Figure 2B; Videos S6 & S7). Once the stimulation lead is placed in the desired location, the guide wire is retracted sufficiently to expose the lead bias that passively stabilizes the lead in the vessel.

### 2.3 Implantation of the sensing lead

Once the stimulation lead is deployed, the sensing lead is implanted into the azygos vein or one of its lateral branches. The sensing lead can be any 6 Fr bipolar lead with a passive fixation bias, based on implanting physician preference. The sensing lead detects respiration by means of transthoracic impedance. The sensing lead is an optional lead. There was no difference in therapy effectiveness or safety outcomes in patients with or without dedicated sensing lead during the pivotal trial. One benefit of a dedicated sensing lead is that remedē System diagnostic software tools may display information with higher respiratory signal quality, which may reduce the number of patient visits needed during therapy titration.

**Figure 3:**
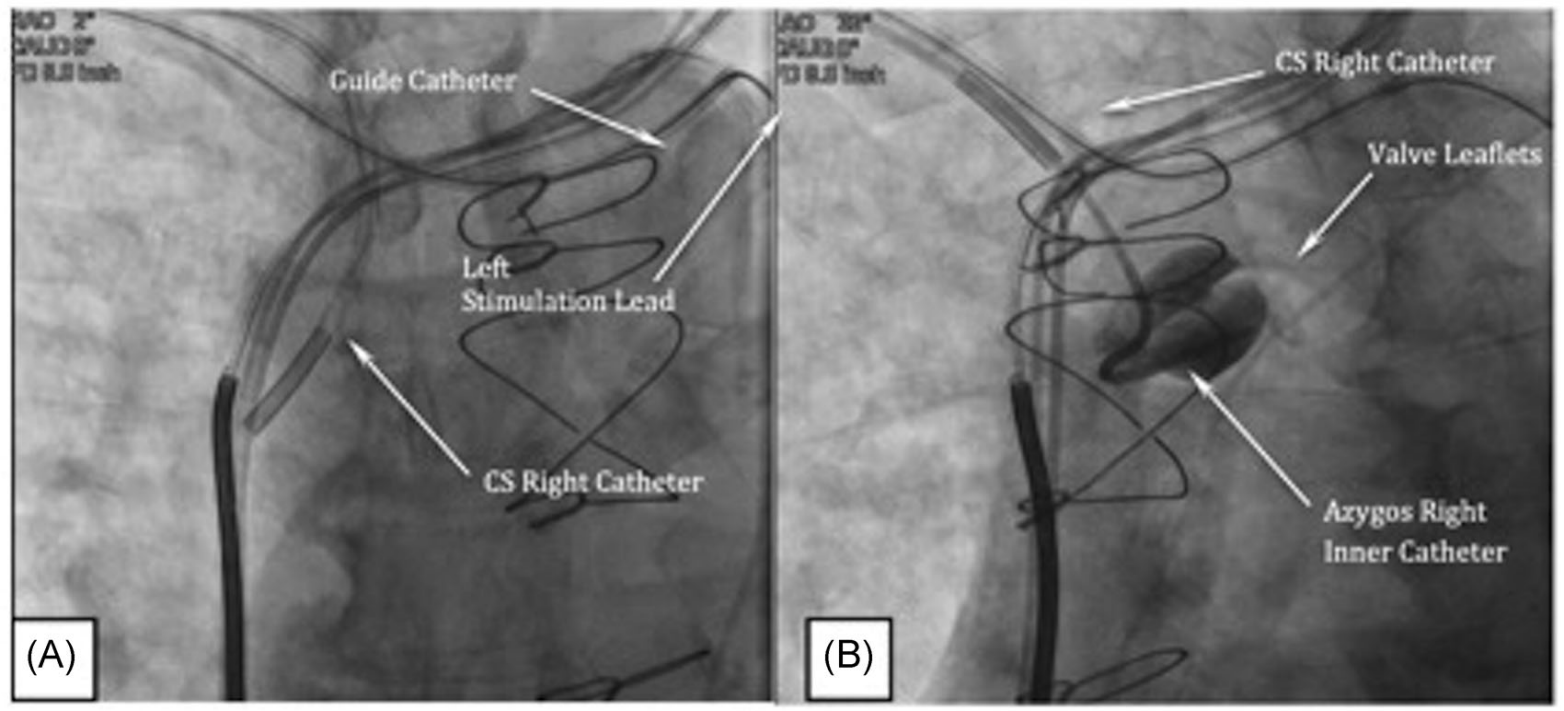
A, Orientation of the coronary sinus right catheter in the superior vena cava to identify the azygos ostium. B, Contrast injection through an inner catheter revealing the azygos ostium and valve leaflets (left anterior oblique)

Using the second venous access wire, a 9 Fr coronary sinus (CS) right guide catheter is delivered into the SVC to a level midway between the tracheal carina and junction of the brachiocephalic veins. In the anterior‐posterior (AP) projection, the tip of the guide catheter is oriented toward the lateral aspect of the SVC (Figure 3A; Video S8). To best visualize the ostium of the azygos vein, the fluoroscope projection should be changed from AP to LAO 30 to 45° or right anterior oblique 30 to 40° and a bolus of contrast delivered. Alternatively, using a 5 Fr Azygous Right inner catheter (eg, Merit medical, Model # 1628‐043), in an LAO projection, the azygos ostium can be visualized with a bolus of contrast delivered with the tip oriented toward the spine (Figure 3B; Video S9). Access to the vein is accomplished by orienting the inner catheter toward the ostium and a 0.035” × 150 cm wire (eg, Glidewire, Terumo Medical, Ann Arbor, MI) is advanced into the azygos vein. The wire is directed around the azygos arch deep into the azygos vein to the level of the diaphragm. A valve is usually encountered in the arch and is frequently traversed by prolapsing the wire tip and employing the same deep breathing or coughing maneuvers described during deployment of the left stimulation lead. The inner catheter is advanced over the wire followed by the 9 Fr guide catheter (Video S10)*.*

**Figure 4:**
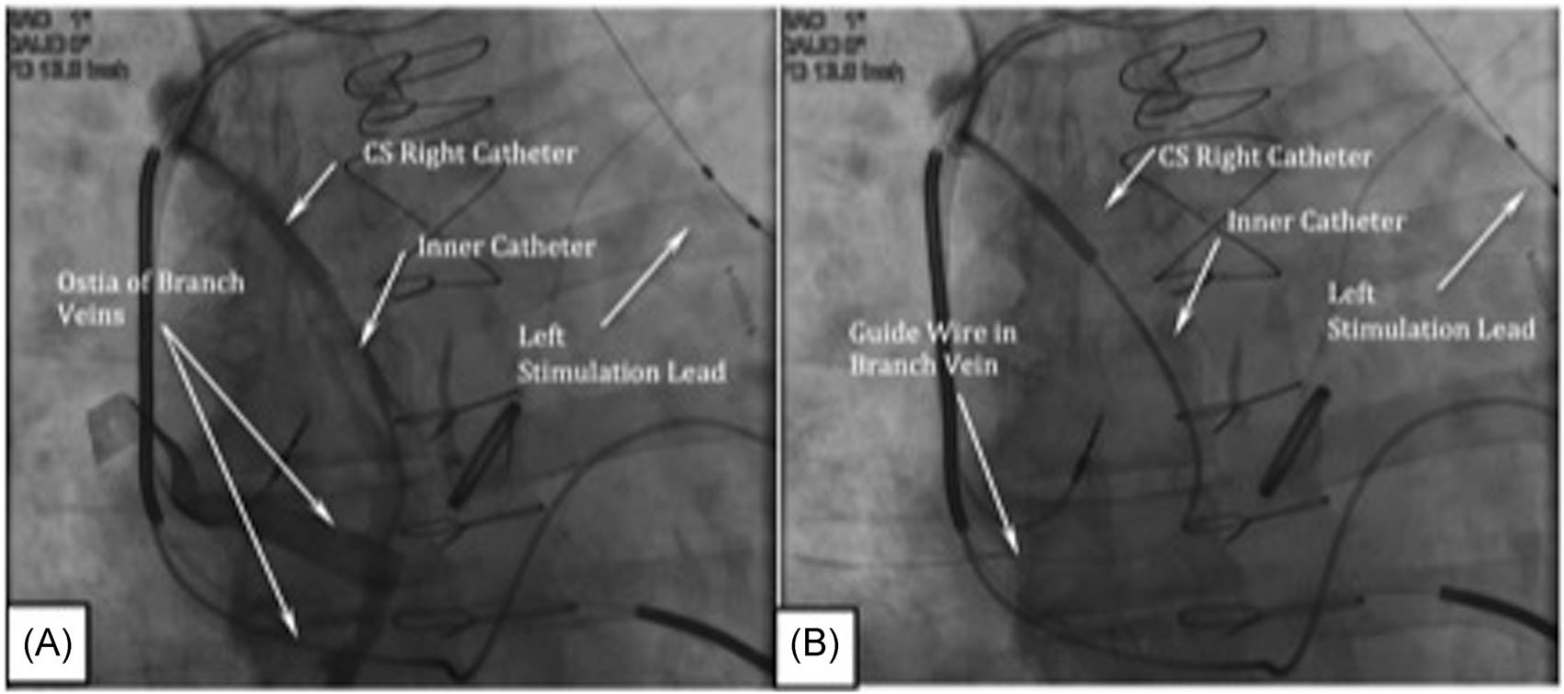
A, Venogram of the azygos vein revealing branch vein ostia. B, Cannulation of the branch vein using a 0.014” coronary wire

**Figure 5:**
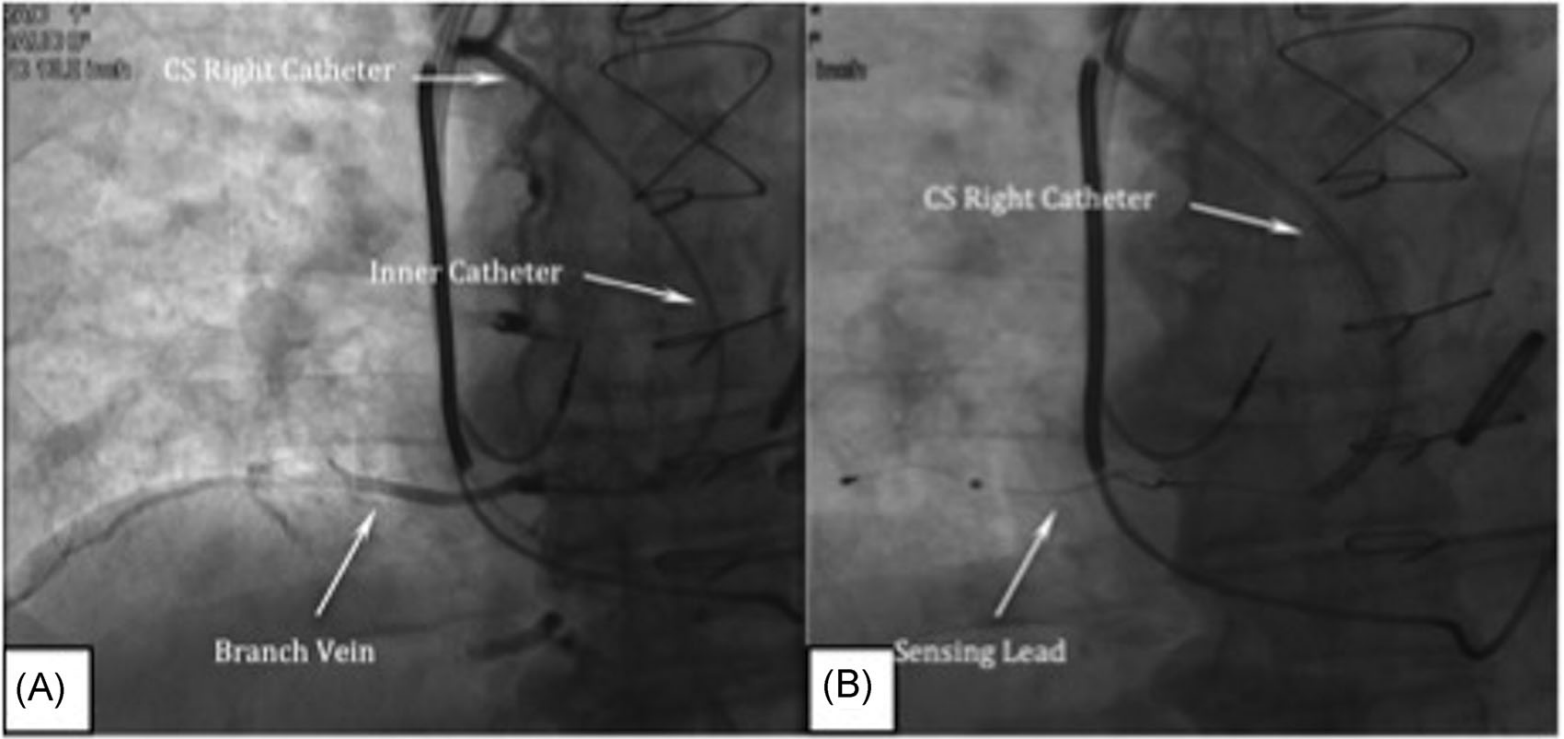
A, Venogram of the target branch off the azygos vein. B, Location of the sensing lead in the branch vein

Once the guide catheter is advanced to a depth midway between the diaphragm and azygos vein ostium, the 0.035” wire is removed and a venogram is performed to confirm cannulation of the azygos vein and to visualize the ostium of the targeted right lateral branch of the vein (Figure 4A; Video S11). The branch vein is cannulated using a new 0.014” coronary wire and an inner catheter. The same inner catheter used during placement of the stimulation lead can be used to subselect the branch off the main tributary of the azygos vein (Figure 4B; Videos S12 & S13). The inner catheter can be used for a selective contrast injection to confirm the course and caliber of the branch vein. (Figure 5A; Video S14). Subsequently, the 9 Fr guide catheter is advanced up to or into the branch in preparation for delivery of the sensing lead. The inner catheter is then removed and the sensing lead is back loaded onto the 0.014” wire and advanced into the side branch, positioning the electrodes 7 to 10 cm lateral to the spine (Figure 5B; Video S15). No electrical testing of the sensing lead is needed. Using the 0.014” coronary wire to stabilize the lead, the guide catheter is removed using a slitter and standard coronary sinus catheter removal techniques. The sensing lead is stabilized using the ligature sleeve.

### 2.4 Testing for adequate phrenic nerve capture

Final positioning of the stimulation lead electrodes at the level of the left atrium occurs before stimulation testing using the phrenic nerve stimulation programmer and external IPG (eIPG; Respicardia, Inc). The programmer is comprised of a touch screen tablet computer and an external programming wand. The wand connects to the programmer via USB and provides a communication link to the CIED. When placed over the IPG the wand uses inductive telemetry for configuration of programmable settings, initiation of system testing, and review of diagnostic data. The eIPG is used for evaluation of stimulation lead placement during implantation. Stimulation testing requires communication with the patient to ensure an appropriate stimulation response. For this reason, general anesthesia is not used during implantation.

**Figure 6:**
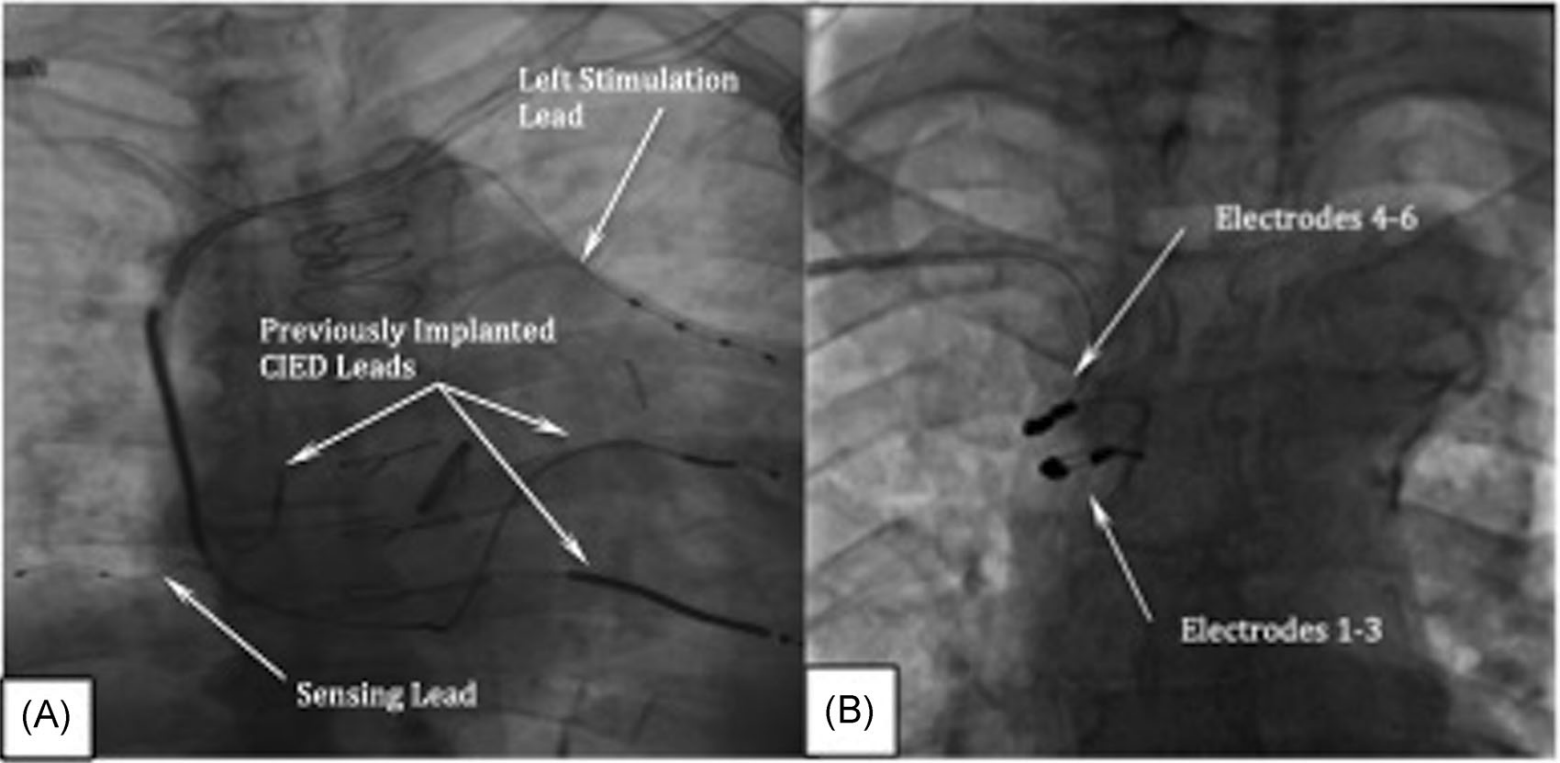
A, Final position of the left stimulation and sensing leads. B, Placement of the right‐sided stimulation lead

Assessment of lead placement and its proximity to the left phrenic nerve is performed by selecting an electrode configuration (cathode–anode electrode pair) and making connections to the eIPG using the test cable. Stimulation lead impedance is measured using the programmer followed by stimulation threshold testing. Delivering single bursts 2 seconds in duration; the stimulation amplitude is incrementally decreased or increased to achieve a moderately strong and full contraction of the diaphragm as determined by means of abdominal palpation or fluoroscopy (Video S16). If complete contraction of the hemidiaphragm cannot be achieved, a new electrode pair is selected, or the stimulation lead is repositioned. If during testing, the patient experiences extra respiratory sensations (ERS), defined as twitching, tingling, or diffuse discomfort in the ipsilateral shoulder or neck either with or without diaphragmatic contraction, another electrode pair is selected or the lead is repositioned. Using 20‐Hz and 150‐microsecond pulse width, 4 mA current or less, typically elicits a moderately strong to strong diaphragmatic contraction. Using the 0.014” coronary wire to stabilize the lead, a slitter is used to cut and remove the catheter. The stimulation lead is stabilized using two manufacturer's ligature sleeves with a recommended minimum 10 cm of stress relieving loop between the sleeves. The IPG and implanted leads are connected using setscrews. The IPG is placed in a subcutaneous pocket in the mid infraclavicular area and secured in place with a single ligature using a suture hole through the IPG header. The final positions of both the left stimulation and sensing leads are shown in Figure 6A; Video S17).

### 2.5 Device activation and operation

After a 1‐month period allowed for healing and lead maturation, patients undergo therapy initiation. Therapy is programmed to automatically begin when the patient is in a sleeping position, at rest during their normal sleep hours, and after lead impedance is measured. Individualized device settings, including therapy start/stop times and programmed stimulation parameters, are determined by in‐office testing, interviewing the patient regarding sleep habits and, when necessary, monitoring patient response to overnight stimulation.

### 2.6 Alternate approach

In some patients, the left stimulation lead was not implanted because of the inability to visualize the PPV, inadequate size of the vessel, the presence of valves, or vessel tortuosity. In these situations, a stimulation lead can be implanted on the right side. Using one of the previously placed venous access wires, an 8 Fr splittable hemostatic introducer is placed into the RBC. A venogram of the RBC and SVC is performed to understand the shape and diameter of the vessel. The respistim R lead is a multipolar, transvenous, stylet‐delivered lead. The proximal end of the lead has IS‐1 terminal pins and the distal end of the lead comprises multiple ring electrodes and a helical shape bias. With the stylet fully advanced to straighten the helical bias, the lead is inserted through the introducer and advanced deep into the SVC; the stylet is then retracted to let the helical bias form within the vessel. The lead is retracted while applying counter‐clockwise rotation until the helices are located in the RBC and upper portion of the SVC and electrodes are oriented on the right lateral aspect of the vessel (Figure 6B). Electrode stability is assessed by instructing the patient to take several deep breaths and make several deep coughs while observing the electrodes using fluoroscopy. If the electrodes appeared unstable, the lead is relocated within the vessel to find the position in which the lead helix is most relaxed and captures the right phrenic nerve. The right stimulation lead is stabilized in the pectoral region using the same methods described for the left stimulation lead.

## 3 RESULTS FROM THE REMEDĒ SYSTEM PIVOTAL TRIAL

Of the 151 randomized patients enrolled in the remedē System Pivotal Trial, 147 (97%) were successfully implanted on the first attempt. The four patients who did not successfully receive an implant had anatomical variability. Ninety‐four (62%) of the 151 patients were implanted with left stimulation leads; 53 (35%) had right stimulation leads implanted. One hundred and five (70%) of the 151 patients were implanted with a sensing lead, 92 out of 94 left lead patients and 16 of the 53 right lead patients. A dedicated sensing lead provided the highest quality sensing signal; implantation was an optional subject to physician discretion. The percentage of patients free from serious adverse events associated with the implant procedure, the system, or the delivered therapy through the 12‐month visit was 91% (95% confidence interval [86%, 95%]).^9^

During implantation, ERS in the ipsilateral shoulder or neck with or without diaphragmatic contraction was detected in 11 of 151 (7%) patients during implantation. All were resolved with repositioning of the lead during the procedure or through the use of alternate electrodes at the time of therapy initiation.

The average overall procedure time (skin to skin) was 2.7 ± 0.8 hours from the first incision to final suture. Implanters with seven or more cases saw a 45‐minute reduction in procedure time as well as decreases in the quantity of fluoroscopy time and contrast volume. Implant duration, fluoroscopy time, and amount of contrast used are summarized in Table 1. Phrenic nerve capture parameters at implant (month 0), 1 month, and 6 months after implant are shown in Table 2.

**Table 1:** Implant procedure data

Variables	Left^a^	Right	Pooled^b^
	(n = 94)	(n = 53)	(n = 151)
Implant duration, h	2.5 ± 0.7 (94)	3.2 ± 0.8 (53)	2.7 ± 0.8 (151)
	2.4 (1.3, 5.3)	3.1 (1.6, 6.2)	2.6 (1.3, 6.2)
Total fluoroscopy, min	33.1 ± 16.6 (93)	59.2 ± 22.2 (53)	43.0 ± 22.5 (150)
	32.0 (4.0, 89.0)	56.0 (27.0, 126.0)	40.0 (4.0, 126.0)
Total contrast, mL^c^	48.2 ± 48.6 (94)	74.8 ± 61.1 (53)	58.2 ± 55.4 (151)
	35 (5, 400)	60.0 (14, 350)	45 (5, 400)
Stimulation lead placement			
Left	100% (94/94)	0% (0/53)	62% (94/151)
None	N/A	N/A	3% (4/151)
Right	0% (0/94)	100% (53/53)	35% (53/151)
Sensing lead implanted	97% (91/94)	26% (14/53)	70% (105/151)

Mean ± SD (n)/median (min, max)

^a^ Patients are counted on the side of the successful lead implanted at the initial implant attempt. Any effort on the opposite side is included as cumulative for the implant attempt.

^b^ Pooled includes four subjects with unsuccessful implant attempts.

^c^ Use of a power injector for administering contrast.

**Table 2:** Capture current soliciting as a strong response

Stimulation lead tested	Visit	n	Mean(mA)	Median(mA)	Standarddeviation (mA)	Minimum(mA)	Maximum(mA)
Left	Implant	94	3.0	3.0	1.3	1.0	7
	1 mo	91	3.0	2.5	1.6	0.9	9
	6 mo	84	2.9	2.5	1.6	0.8	9
Right	Implant	53	6.6	7.0	1.9	3.5	10
	1 mo	51	5.6	5.5	2.3	2.0	10
	6 mo	47	5.3	5.0	2.5	1.7	10

A concomitant CIED was previously implanted in 64 (47%) patients. Patients with concomitant CIEDs undergo testing at the time of implant. During the trial, there was one shock delivered by an implantable cardiac defibrillator because of oversensing, which was resolved by reprogramming of the ICD. This incident resulted in changes in the testing procedure. There were no incidents of pacing inhibition.

## 4 DISCUSSION

The remedē System implant procedure was done by electrophysiologists and previously shown to be safe, in part, because of the similarity of the procedure to contemporary methods and techniques used to implant CIEDs. While the anatomical targets for lead placement are distinct from those of CIEDs, the size and type of guide wires, and the type and functionality of the catheters are the same as those used for implanting a biventricular CIED. The techniques and means for navigation of the lead (eg, advancing the lead while retracting the wire) to the new anatomical locations are similarly familiar to electrophysiologists experienced in biventricular CIED implants. A procedure‐related complication rate of 6% is similar to the 8.6% left ventricular lead‐related complications occurring in patients with biventricular CIEDs at a similar stage of development and 4% perioperative complications later reported in the REsyncronization reVErses Remodeling in Systolic left vEntricular dysfunction trial.^14^, ^15^ While there was one venous dissection associated with insertion of the catheter in the subclavian vein, this AE did not occur with cannulation of the PPV or brachiocephalic veins and resolved without sequelae.

The energy delivered by the phrenic nerve stimulator is different from that of a CIED. The output applied to the phrenic nerve is a burst of pulses (60‐300–microsecond pulse width at 10‐40 Hz frequency) having varying amplitude that produce a smooth diaphragmatic contraction similar to that occurring with a normal breath, not the “hiccup” response that occurs when a left ventricular lead of a cardiac resynchronization device stimulates the phrenic nerve. The recurring smooth diaphragmatic contraction enables the resumption of a normal breathing pattern and stabilization of blood carbon dioxide levels.^16^ By stabilizing carbon dioxide, phrenic nerve stimulation prevents apneic events and the subsequent periods of rapid breathing. Sensing of this respiration pattern is accomplished by programming a sensing vector using either the stimulation lead or sensing lead. The vector is determined at therapy initiation at which time various sensing vectors are evaluated.

Compared to the surgical implantation of a cuff electrode, the transvenous approach eliminates the risk of damage to the nerve, cuff‐related neural compression, and impairment of blood flow that can lead to degeneration and demyelination of axons. Furthermore, the transvenous approach offers the patient a rapid and comfortable recovery. The mean procedure time of 2.7 hours and the improvement in procedure time with growing experience commensurate with that previously reported.^17^ The practicality and safety of implanting the remedē system permits the delivery of the only therapy that to date has been proven in a randomized controlled trial to ameliorate CSA and mitigate its harmful consequences.^8^

## 5 CONCLUSION

Phrenic nerve stimulation is a safe and novel means for the treatment of CSA. The transvenous implantation technique of the remedē System is very similar to CIED procedures utilizing instruments and techniques familiar to electrophysiologists. The ability to implant the remedē System enables the only therapy shown to meaningfully improve CSA and its detrimental clinical sequelae.

## CONFLICT OF INTERESTS

Dr Augostini has relationships with commercial interests in the following areas: Consultant/Speaker Bureau – Respicardia; and Advisory Board Membership.

## Supporting information

Supporting information

Supporting information

Supporting information

Supporting information

Supporting information

Supporting information

Supporting information

Supporting information

Supporting information

Supporting information

Supporting information

Supporting information

Supporting information

Supporting information

Supporting information

Supporting information

Supporting information

Supporting information
