# Supplementary material for: How to implant a phrenic nerve stimulator for treatment of central sleep apnea?
Source: J Cardiovasc Electrophysiol. 2019 Mar 18;30(5):792–9. doi: 10.1111/jce.13898 (PMC6850096; doi:10.1111/jce.13898)
Supplement: Supplementary file 18 — Supporting information [file JCE-30-792-s018.docx]

Supplementary video 1: Advancing the guide catheter over a J wire into the left brachiocephalic vein

Supplementary video 2: Examining the left brachiocephalic vein with puff of contrast

Supplementary video 3: Non-selective contrast injection in the left brachiocephalic vein

Supplementary video 4: Cannulation of the pericardiophrenic vein using the 0.014” wire

Supplementary video 5: Advancing the wire deeper into pericardiophrenic vein

Supplementary video 6: Advancing the stimulation lead into pericardiophrenic vein

Supplementary video 7: Positioning the stimulation lead within the pericardiophrenic vein.

Supplementary video 8: Advancing the second guide catheter into SVC

Supplementary video 9: Non-selective and selective contrast injection to identify the azygos vein

Supplementary video 10: Cannulation of the azygos vein with the sub-selection catheter and guide catheter

Supplementary video 11: Advancing a sub-selection catheter into the azygos vein

Supplementary video 12: Non-selective contrast injection to identify a right lateral branch of the azygos vein

Supplementary video 13: Cannulation of the right lateral branch of the azygos vein

Supplementary video 14: Selective contrast injection of the right lateral branch of the azygos vein

Supplementary video 15: Positioning the sensing lead in the right lateral branch of the azygos vein

Supplementary video 16: Full contraction of the diaphragm from stimulation of the phrenic nerve

Supplementary video 17: Final location of the IPG and leads
